# Supplementary material for: Direct Cryo-ET observation of platelet deformation induced by SARS-CoV-2 spike protein
Source: Nat Commun. 2023 Feb 4;14:620. doi: 10.1038/s41467-023-36279-5 (PMC9898865; doi:10.1038/s41467-023-36279-5)
Supplement: Supplementary file 1 — Supplementary Information [file 41467_2023_36279_MOESM1_ESM.pdf]

Supplemental Information

Direct Cryo-ET observation of platelet deformation induced by SARS-CoV-2 Spike protein

Christopher Cyrus Kuhn[1], Nirakar Basnet[1*], Satish Bodakuntla[1*], Pelayo Alvarez-Brecht [1,2*], Scott Nichols[1], Antonio Martinez-Sanchez[2,5], Lorenzo Agostini[1], Young-Min Soh[1], Junichi Takagi[3], Christian Biertümpfel[1], Naoko Mizuno[1,4]

[1] Laboratory of Structural Cell Biology, National Heart, Lung, and Blood Institute, National Institutes of Health, 50 South Dr., Bethesda, MD, 20892, USA

[2] Department of Computer Sciences, Faculty of Sciences - Campus Llamaquique, University of Oviedo, Oviedo 33007, Spain

[3] Osaka University Institute for Protein Research, Osaka University, 3-2 Yamadaoka, Suita, Osaka 565-0871, Japan

[4] National Institute of Arthritis and Musculoskeletal and Skin Diseases, National Institutes of Health, 50 South Dr., Bethesda, MD, 20892, USA

[5]Health Research Institute of Asturias (ISPA), Avenida Hospital Universitario s/n, 33011, Oviedo, Spain

*equally contribution

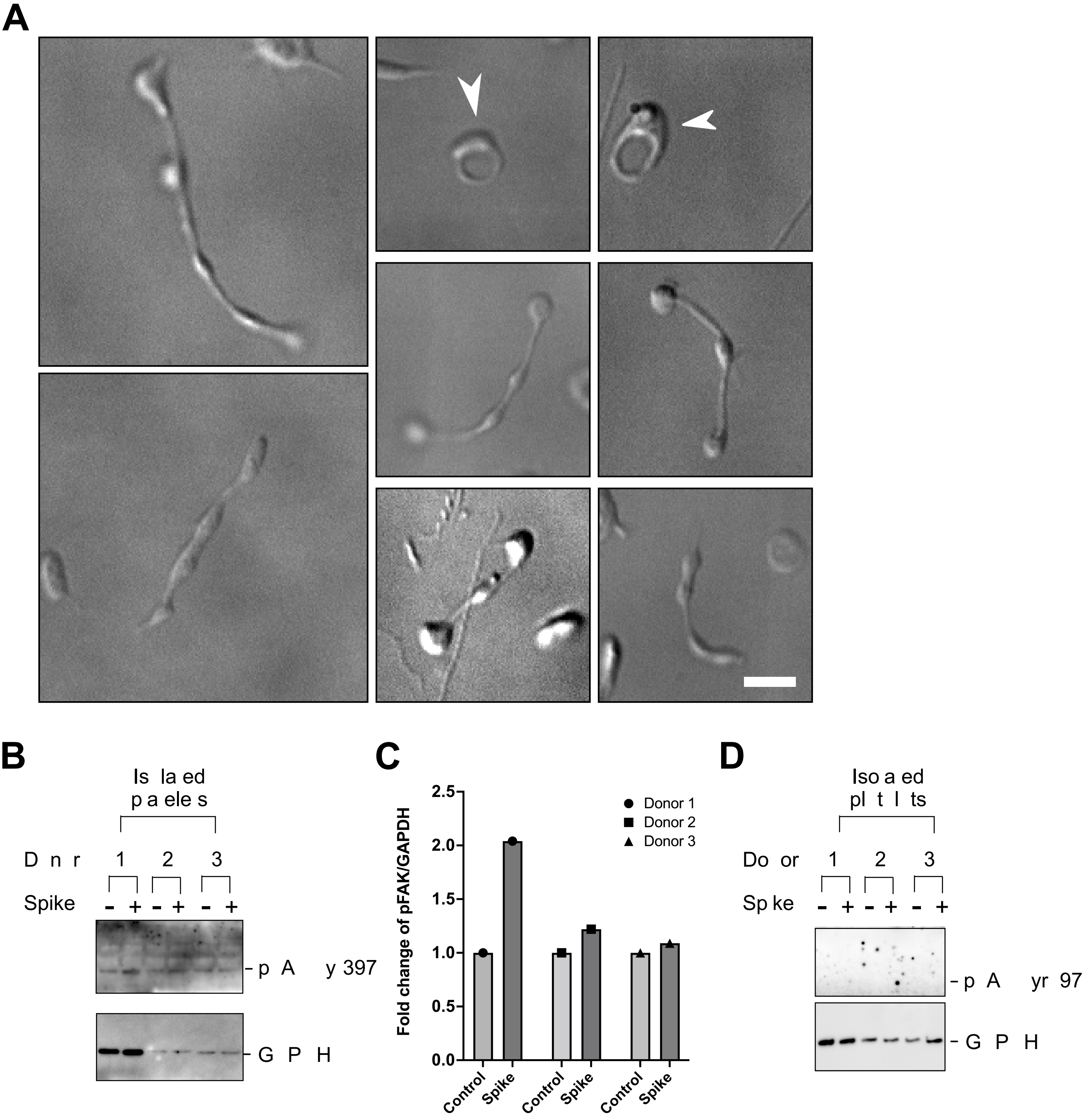

Supplementary Figure 1. Platelets incubated with SARS-CoV-2 S protein reveal proplatelet-like morphologies.

(A) A subset of platelets showed a tubular appearance with multiple globular bodies along their elongated shape after the incubation with S protein. Some platelets had a ring-shaped appearance in the presence of the S protein. (B) The adherent platelets were collected in RIPA lysis buffer and the total concentration was quantified with Bradford assay. 30 µg of total protein was loaded on the gel and probed against pFAK or GAPDH. (C) Quantification of pFAK in adherent platelets normalized against GAPDH concentrations, showing the values of 2.04 (donor 1), 1.22 (donor 2) and 1.09 (donor 3). (D) The floating platelets were handled as specified in (B) and probed against pFAK or GAPDH. Scale bar: (A) = 5 µm.

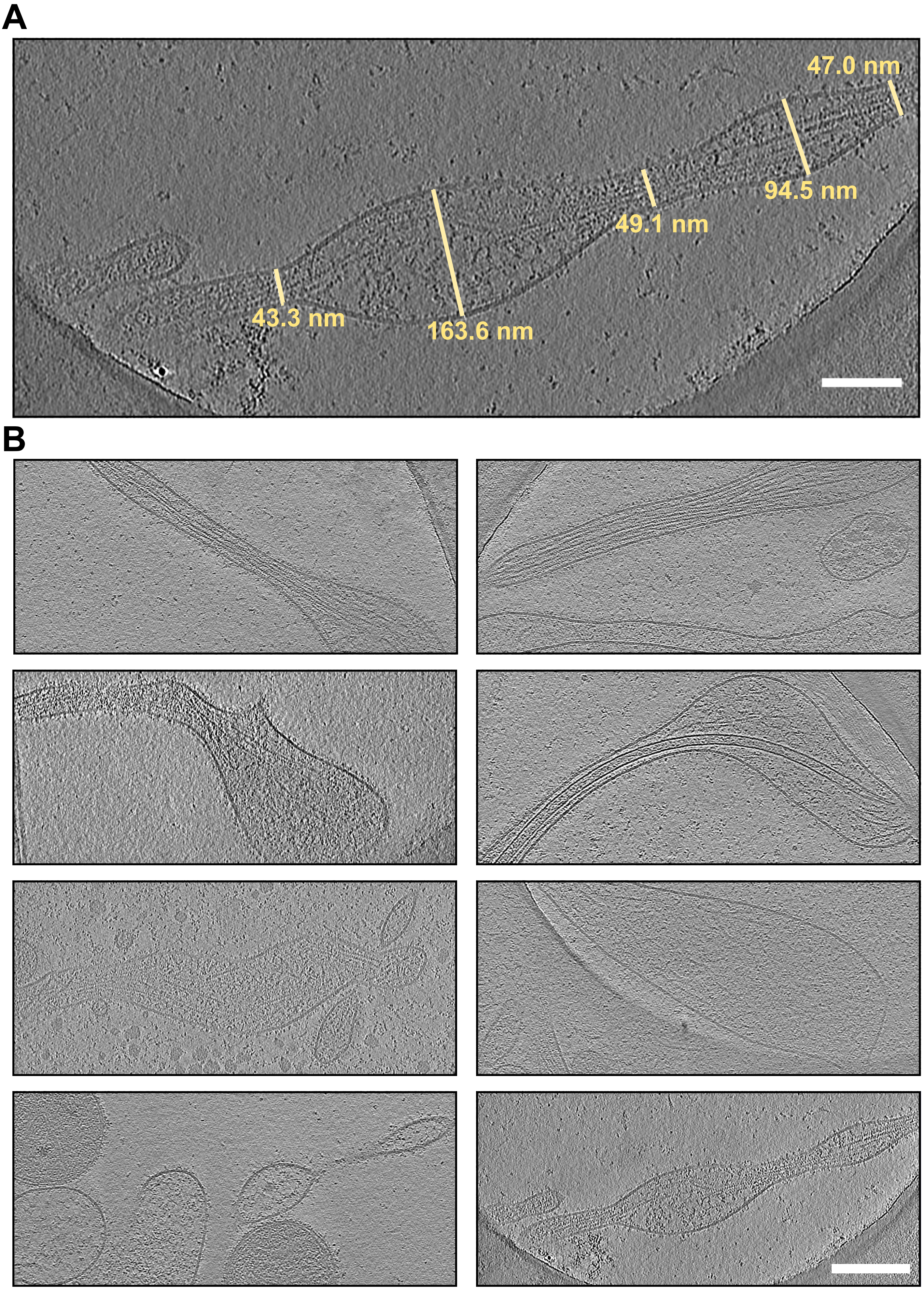

Supplementary Figure 2. Cryo-electron tomographic visualization of platelets incubated exposed to SARS CoV 2 S protein.

(A) Filopodia width of the platelet incubated with S protein. (B) Central Slices of analyzed tomograms. The images show a slice through the deconvoluted tomogram used for further analysis. Scale Bars: (A) = 100 nm; (B) = 200 nm

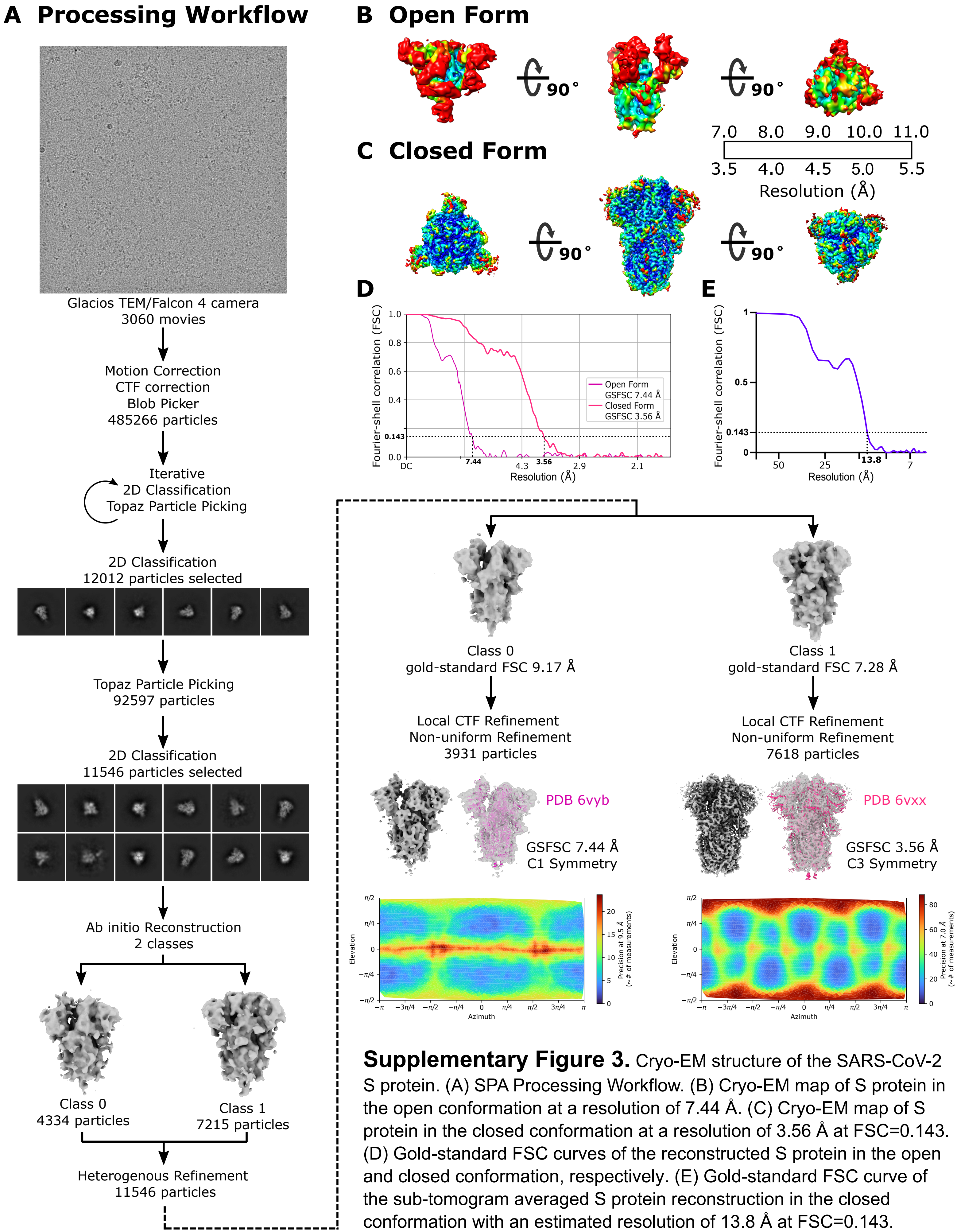

Supplementary Figure 3. Cryo-EM structure of the SARS-CoV-2 S protein. (A) SPA Processing Workflow. (B) Cryo-EM map of S protein in the open conformation at a resolution of 7.44 Å. (C) Cryo-EM map of S protein in the closed conformation at a resolution of 3.56 Å at FSC=0.143. (D) Gold-standard FSC curves of the reconstructed S protein in the open and closed conformation, respectively. (E) Gold-standard FSC curve of the sub-tomogram averaged S protein reconstruction in the closed conformation with an estimated resolution of 13.8 Å at FSC=0.143.

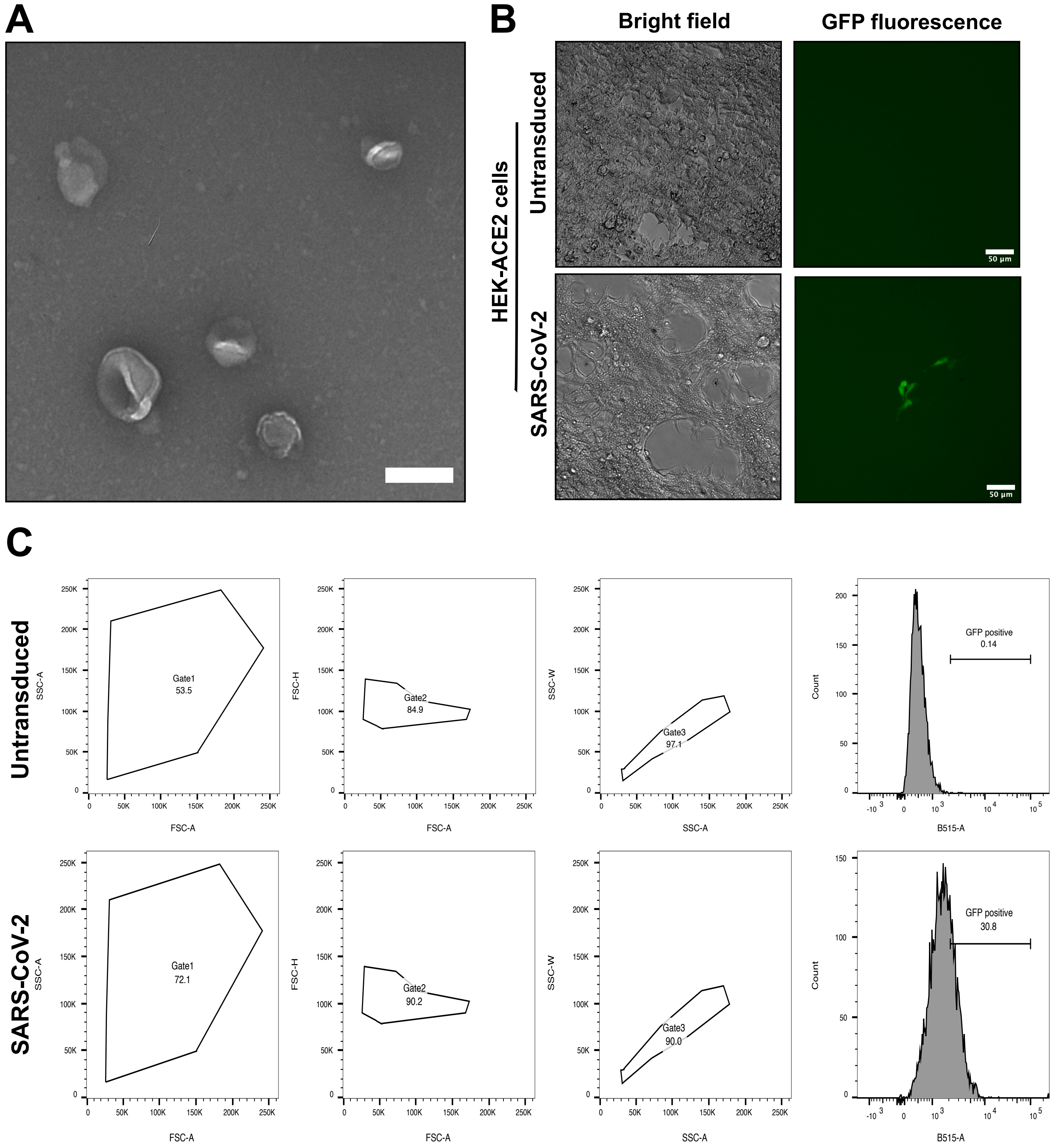

Supplementary Figure 4. Characterization of SARS-CoV-2 S-pseudotyped lentiviral particles.

(A) Negative-staining EM with 2% uranyl acetate of SARS-CoV-2 S-pseudotyped lentiviral particles. (B) Light microscopic images of HEK-hACE2 cells with or without treatment with pseudotyped lentivirus encoding ZsGreen. (C) Flowcytometry analysis of HEK-hACE2 cells transduced with pseudotyped lentivirus encoding ZsGreen backbone plasmid. The plot shows percentage of green-fluorescent cells with or without the incubation of S-pseudotyped lentivirus with HEK-hACE2 cells. Gating was first applied to select the live cells and then the gate in histogram was set such that the uninfected cells show less than 1% positive cells, and the same gate has been applied to infected cells. Scale bars: (A) = 100 nm; (B) = 50 μm.

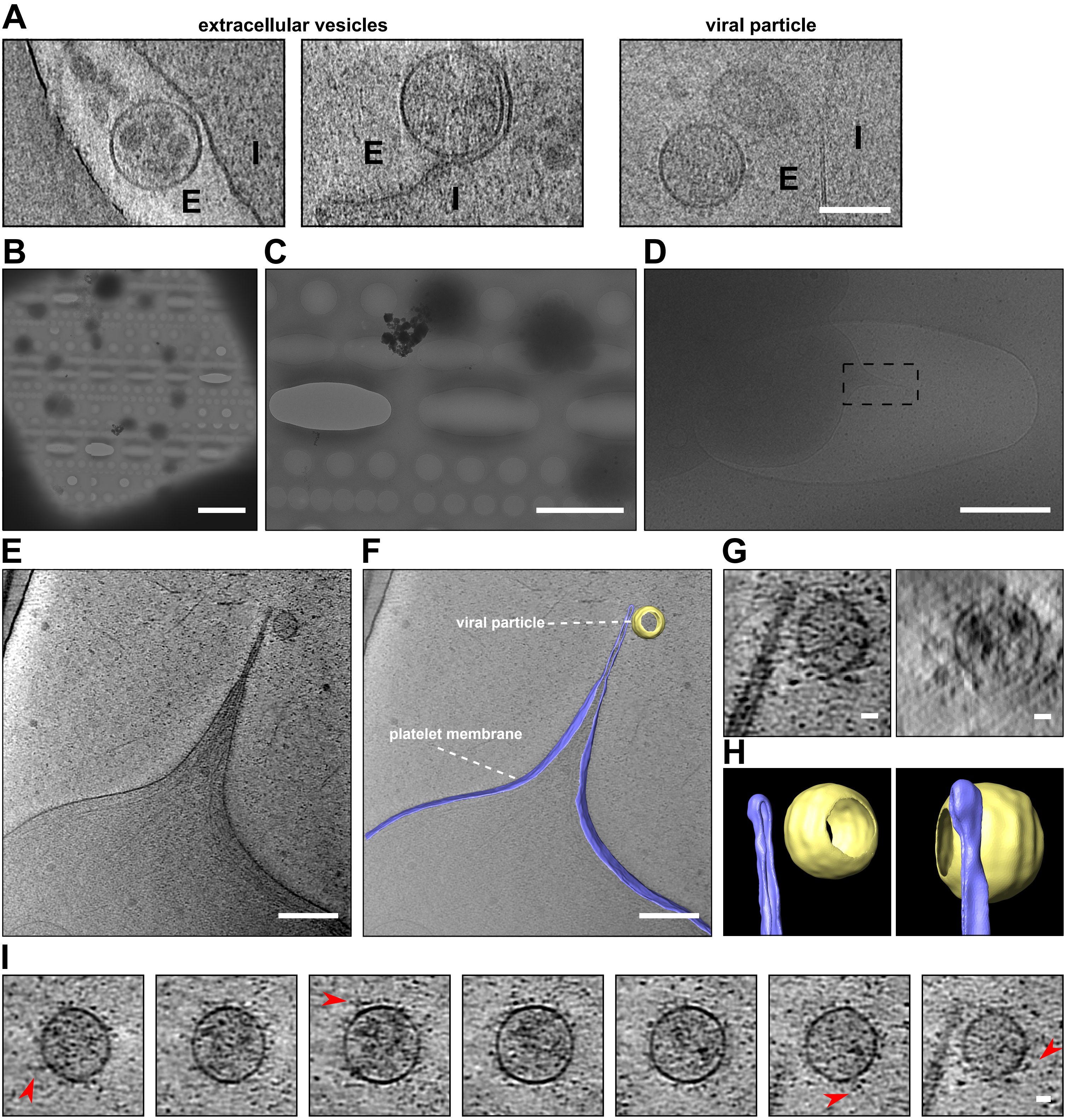

Supplementary Figure 5. SARS CoV 2 S pseudotyped viral particles on platelet plasma membrane by cryo-ET.

(A) Extracellular vesicles and S protein pseudotyped virus under cryo-EM condition. (E: extracellular, I: intracellular). (B) Overview of grid square of platelets incubated with pseudotyped viral particles. (C) Low magnification of platelets observed in the presence of pseudotyped viral particles. (D) Platelet with pseudotyped viral particle exhibits the formation of a filopodial protrusion. The dashed box indicates the area of tomogram data collection. (E) Slice of the reconstructed tomogram showing a virus-like particle on the platelet plasma membrane. (F) Segmentation of the tomogram with virus-like particle at the platelet plasma membrane (purple – platelet plasma membrane, yellow – virus-like particle membrane). The tomogram lacks top and bottom due to the "missing wedge" effect of tomographic data collection. (G) Zoom-in views on the contact site of platelet and virus-like particle. (H) Zoom-in view on the segmented platelet membrane and virus-like particle membrane (purple – platelet plasma membrane, yellow – virus-like particle membrane) (I) Slices thorough the virus-like particle at the filopodia tip. Red arrows point at the protein densities on the membrane surface. Scale bars: (A)=100nm; (B)=10 μm; (C)= 5 μm; (D)=1 μm; (E)&(F)=200 nm; (G)&(I)=20nm.

Supplementary Table 1

Cryo-EM/Cryo-ET data collection, refinement and validation statistics

	S protein (SPA) (EMDB-26798)	Platelet with S protein #1 (tomography) (EMDB-26794)	Platelet with S protein #2 (tomography) (EMDB-26796)
**Data collection and processing**			
Magnification	150,000	33,000	33,000
Voltage (kV)	200	300	300
Electron exposure (e–/Å^2)	49	123	123
Defocus range (μm)	-0.8 to -2.4	-3 to -5	-3 to -5
Pixel size (Å)	0.93	2.76	2.76
Symmetry imposed	C3	NA	NA
Initial particle images (no.)	11,549	NA	NA
Final particle images (no.)	7618	NA	NA
Map resolution (Å)	3.56	NA	NA
FSC threshold	0.143		
Map resolution range (Å)			
