# Supplementary material for: Direct Cryo-ET observation of platelet deformation induced by SARS-CoV-2 spike protein
Source: Nat Commun. 2023 Feb 4;14:620. doi: 10.1038/s41467-023-36279-5 (PMC9898865; doi:10.1038/s41467-023-36279-5)
Supplement: Supplementary file 2 — Description of Additional Supplementary Files [file 41467_2023_36279_MOESM2_ESM.pdf]

Description of Additional Supplementary Files

File Name: Supplementary Movie 1
Description: **Representative DIC time lapse movies of platelets with or without SARS-CoV-2 S protein. (**A) Movies of platelets seeded on collagen type I coating preincubated without (left) or with S protein (right). (B) Movies of platelets seeded on poly-lysine coating preincubated without (left) or with S protein (right). (C) Movies of platelets seeded on fibronectin coating preincubated without (left) or with S protein (right). Scale bars: 5 µm.

File Name: Supplementary Movie 2
Description: **Reconstructed tomograms, acquired on platelets incubated with SARS-CoV-2 S protein. (**A) Tomographic reconstruction of a filopodial platelet protrusion. (B) Tomographic reconstruction of a platelet protrusion including a microtubule. Scale Bars: (A) 100 nm; (B) 200 nm.
